# The mediating effect of positive self-belief on the relationship between work stress and humanistic nursing competence among Chinese nurses: a cross-sectional study

**DOI:** 10.1186/s12912-026-04468-4

**Published:** 2026-02-28

**Authors:** Lu Sun, Huixia Cui, Qingyun Li, Wenlong Tang, Qianya Zhang, Rong Mao, Na Xu

**Affiliations:** School of Nursing, Wannan Medical College, Wuhu, 241003 China

**Keywords:** Clinical nurses, Work stress, Positive self-belief, Humanistic nursing competence

## Abstract

**Background:**

Humanistic nursing competence constitutes not only the core competency of nurses but also their professional integrity and service ethos. This study aimed to investigate the mediating role of positive self-belief in the relationship between nurses’ work stress and humanistic nursing competence.

**Methods:**

This cross-sectional study employed convenience sampling to recruit 542 clinical nurses from hospitals of varying tiers across 10 cities in Anhui Province. The participants were assessed via the Nurse Work Stress Scale, the Oxford Positive Self-Belief Scale, and the Healthcare Professional Humanization Scale for Nursing. Following model construction, path analysis was conducted via AMOS 26.0 software.

**Results:**

The humanistic nursing competence score for clinical nurses was 69.83 ± 11.47. Correlation analysis revealed that clinical nurses’ work stress was moderately negatively correlated with humanistic nursing competence (*r* = -0.426, *P* < 0.001), while positive self-belief was strongly positively correlated with humanistic nursing competence (*r* = 0.629, *P* < 0.001). Additionally, work stress was moderately negatively correlated with positive self-belief (*r* = -0.460, *P* < 0.001). A mediational effect analysis revealed that the total effect of work stress on clinical nurses’ humanistic nursing competence was − 0.155, with a direct impact of -0.041 accounting for 26.67% of the total effect. Positive self-belief partially mediated the relationship between clinical nurses’ work stress and humanistic nursing competence, with a mediating effect of -0.113, accounting for 73.33% of the total effect.

**Conclusions:**

The humanistic nursing competence of clinical nurses in Anhui Province is moderately weak. Work pressure not only directly impacts humanistic nursing competence but also indirectly influences it through positive self-beliefs. Nursing administrators should implement evidence-based, multidimensional intervention strategies. These include conducting structured positive self-belief group training, optimizing nurse staffing allocation, incorporating humanized care capabilities into the core quality evaluation system, and incorporating positive psychology curricula into nursing education. This systematic approach will enhance nurses’ humanistic care capabilities.

**Clinical trial number:**

Not applicable.

## Background

Humanistic nursing, as a patient-centered care model focused on patients’ physical and mental well-being, rights, and needs, has become a key indicator for measuring nursing quality worldwide [1–2]. Classical theories of caring, such as Watson’s Theory of Human Caring [3] and Swanson’s Caring Model [4], further deepened its inner meaning, indicating that humanistic nursing is not the mechanical execution of tasks but rather a professional practice that relies heavily on nurses’ intrinsic motivation, emotional investment, and the establishment of meaningful nurse-patient relationships [5]. It requires nurses to provide holistic care encompassing physical, mental, and emotional dimensions, grounded in respect for each patient’s unique individuality and overall life circumstances [6–7]. Extensive research confirms that higher levels of humanistic nursing competence not only enhance patients’ treatment adherence, sense of security, and satisfaction [8–9], but also strengthen nurses’ own professional identity, sense of accomplishment, and well-being by fostering meaningful nurse-patient relationships [9–11].

However, the high work stress prevalent in clinical settings severely exhausted the emotional and cognitive resources nurses require to deliver humanistic care, posing a significant challenge to their capabilities [12–14]. In the context of China’s high-pressure healthcare environment, nurses commonly face multiple stressors, including child-rearing responsibilities, demanding workloads, frequent night shifts, complex nurse-patient relationships, emergency incident management, and occupational exposure risks [15]. Prolonged exposure to high-intensity work stress not only compromises nurses’ physical and mental health and career development, leading to occupational burnout, decreased job satisfaction, and heightened turnover intentions [12–16], but may also erode the intrinsic psychological motivation essential for delivering humanistic care.

Although previous studies have demonstrated a negative correlation between nurses’ work stress and their humanistic nursing competence [17–18] and explored the mediating roles of external or specific psychological resources such as social support [19] and psychological capital [20], these investigations have yet to uncover the deep psychological mechanisms through which work stress influences humanistic nursing competence from the perspective of intrinsic motivation. Therefore, the core question of how work stress influences nurses’ humanistic care capabilities through the psychological processes of intrinsic motivation remains an urgent area for research.

To elucidate the mechanism, this study employs self-determination theory as its core theoretical framework. This theory [21], proposed by psychologists Edward L. Deci and Richard M. Ryan in the late 1970s, is a motivational process theory concerning human self-determined behavior. This theory posits that the key to individual behavior and development lies in the fulfillment of three fundamental psychological needs: autonomy, competence, and relatedness. When the social environment supports these needs, individuals can function at their best, maintaining a positive psychological state and producing more positive behavioral outcomes. Conversely, an obstructive social environment impedes the fulfillment of these needs, thereby weakening an individual’s intrinsic motivation and positive behaviors. In this study, the high work stress faced by nurses constitutes an obstructive social environment. It impedes the fulfillment of basic psychological needs by imposing excessive workloads, restricting professional autonomy, and crowding out the time and energy required to build nurse-patient relationships. Persistent frustration of these needs may deplete the intrinsic motivation necessary for providing humanistic care.

Building upon this foundation, this study proposes the concept of positive self-belief as a key mediating variable in elucidating the process, positing it as the crucial psychological link connecting work stress and the capacity for humanistic care. In this study, positive self-belief can be regarded as an integrated psychological resource formed at the self-cognitive and emotional levels based on the degree to which an individual’s fundamental psychological needs are met. It transcends the perception of needs within specific contexts. Positive self-belief, as a stable and positive integrative psychological representation of one’s professional value, capabilities, and developmental potential, organically integrates emotion, cognition, and self-consistency, profoundly influencing an individual’s feelings, thoughts, and actions [22]. In high-pressure work environments, persistent frustration of psychological needs gradually erodes nurses’ positive self-beliefs. This undermining of fundamental beliefs further weakens the intrinsic motivation and behavioral foundation for nurses to deliver humanistic care. Research in positive psychology also indicates [23] that establishing a positive self-perception within the field of psychology is a crucial factor in developing mental well-being. Based on this, we propose the following research hypothesis: In China’s high-workload, low-control healthcare settings, nurses’ high work stress acts as an obstructive social environment. It undermines their positive self-belief—a source of intrinsic motivation—by damaging fundamental psychological needs, ultimately negatively impacting their capacity for humanistic nursing.

This study aims to examine the mediating pathway of “work stress → positive self-belief → humanistic nursing competence” based on self-determination theory, thereby elucidating the underlying psychological mechanisms through which Chinese nurses maintain or lose their humanistic nursing competence under high-pressure environments. It also tests the following hypotheses: 

### H1

Work stress is negatively correlated with positive self-belief (Path a).

### H2

 Work stress is negatively correlated with humanistic nursing competence (Path c).

### H3

Positive self-belief is positively correlated with humanistic nursing competence (Path b).

### H4

 Positive self-belief mediates between work stress and humanistic nursing competence (Path c’).

## Methods

### Research design

This study employed a cross-sectional survey method to examine the relationships among three variables: nurses’ work stress, positive self-belief, and humanistic nursing competence. The implementation and documentation of the study strictly adhered to the Standardized Reporting of Observational Studies in Epidemiology (STROBE) guidelines.

### Participants and sample

This study employed convenience sampling to select 542 clinical nurses from medical institutions across 10 prefecture-level cities in Anhui Province as research subjects. The inclusion criteria were as follows: (1) held a valid nurse practice license; (2) were currently employed at a hospital; (3) had at least one year of professional work experience; and (4) signed an informed consent form and voluntarily participated in the study. The exclusion criteria were as follows: (1) intern nurses or student nurses; (2) personnel on leave during the survey period.

Based on the principle that the sample size should be ≥ 200 according to structural equation modeling [24–25] and considering a 10% sample nonresponse rate, the minimum sample size was calculated to be 220. After fully considering model complexity and controlling for sampling error and potential invalid responses, a total of 563 questionnaires were distributed. Following the exclusion of 21 noncompliant questionnaires via the “list deletion method,” 542 valid samples were ultimately included, resulting in an effective response rate of 96.3%.

### Evaluation methods and tools

#### Sociodemographic data questionnaire

This encompasses nine items: nurses’ gender, age, working age, professional title, marital status, educational qualification, hospital tier, work location, and current department.

#### Nurses’ work stress sources scale

The Nursing Work Stress Sources Scale [26], developed by Chinese scholars including Xiaomei Li in 2000, assesses nurses’ current work stress levels and their sources. It comprises five dimensions with 35 items: Nursing Profession and Work (7 items, possible range: 7–28 points), Workload and Time Management (5 items, possible range: 5–20 points), Work Environment and Resources (3 items, possible range: 3–12 points), Patient Care (11 items, possible range: 11–44 points), and Management and Interpersonal Relationships (9 items, possible range: 9–36 points). All the items are phrased as positive statements and are answered on a 4-point Likert scale, with options ranging from “Strongly Disagree” (1 point) to “Strongly Agree” (4 points). The total score ranges from 35 to 140 points, with higher scores indicating greater occupational stress levels. This scale has a broad application base in Chinese nursing research, with a Cronbach’s alpha coefficient of 0.981 [26]. In this study, the Cronbach’s alpha coefficient for the scale was 0.948.

#### The oxford positive self scale

Professor Daniel developed this scale in the UK in 2022 [27]. The version used in this study is the Chinese translation previously conducted by the research team [28], which has undergone an expert content validity review. The scale comprises four dimensions with 24 items: Mastery Dimension (7 items, possible range: 0–28 points), Strength Dimension (8 items, possible range: 0–32 points), Enjoyment Dimension (5 items, possible range: 0–20 points), and Character Dimension (4 items, possible range: 0–16 points). It employs a 5-point Likert scale ranging from 0 to 4, corresponding to “strongly disagree” to “strongly agree.” The total score ranges from 0 to 96 points (sum of all items), with higher scores indicating greater levels of positive self-belief. The Chinese version has good reliability and validity, with a Cronbach’s alpha coefficient of 0.935 [28]. In this study, the Cronbach’s alpha coefficient for the scale was calculated as 0.893.

#### The healthcare professional humanization scale (HUMAS) for nursing

Developed by Pérez-Fuente et al. [7], this scale was adapted into Chinese by the Chinese scholar Li Kangyuan [29]. It comprises 19 items across 5 dimensions: Disposition to Optimism Dimension (3 items, possible range: 3–15 points), Sociability Dimension (3 items, possible range: 3–15 points), Emotional Understanding Dimension (3 items, possible range: 3–15 points), Self-Efficacy Dimension (5 items, possible range: 5–25 points), and Affection Dimension (5 items, possible range: 5–25 points). It employs a 5-point Likert scale, with responses ranging from “never” to “always” scored as 1 to 5 points, respectively. The total score ranges from 19 to 95 points (sum of all items), with higher scores indicating stronger humanistic nursing competencies among nurses. The Chinese version of the scale yielded a Cronbach’s alpha coefficient of 0.851 [29]. In this study, the Cronbach’s alpha coefficient for the scale was calculated as 0.906.

### Study settings and data collection

The research data were collected between January 2025 and March 2025. This study surveyed nurses across hospitals of varying grades in 10 prefecture-level cities within Anhui Province, which is in eastern China. The nursing staff surveyed encompassed four educational levels: secondary technical school, junior college, undergraduate, and master’s degrees or above, and three professional titles: junior, intermediate, and senior. Following approval from the School Ethics Committee of Wannan Medical College, data collection commenced. Before conducting the survey, we contacted the nursing management experts of relevant hospitals to secure their support. We provided the participating nurses with detailed explanations of the survey process, objectives, and significance. After signing the electronic informed consent form, the nurses voluntarily completed the anonymous data collection by scanning a QR code for the electronic questionnaire created via the Chinese electronic platform Questionnaire Star. Researchers extracted completed questionnaires from the backend and rigorously reviewed response quality. Any questionnaire exhibiting ten consecutive identical selections or showing obvious repetitive patterns was deemed invalid and excluded.

### Ethical considerations

This study was approved by the Ethics Committee of Wannan Medical College (Ethics Approval Number: 2025003). The study was conducted in accordance with the Declaration of Helsinki. All the participants engaged in the survey anonymously and have signed electronic informed consent forms (Before beginning the questionnaire, the participants first accessed a mandatory informed consent page. This page clearly and comprehensively outlines core information, including the study’s purpose, procedures, risks and benefits, confidentiality provisions, and the principle of voluntary participation. The participants had to read all the content and manually check the box stating “I have read and understood the above information and voluntarily agree to participate in this study” before clicking the button to proceed to the formal questionnaire). We assured participants that their involvement in this study would not adversely affect their relationship with their employing hospital. To ensure data security and confidentiality, all collected data were processed in an anonymous format and stored on password-protected, institutionally encrypted computers. Only core research team members have authorized access to these data, particularly for this study. Furthermore, participants may withdraw or terminate their participation at any time during the study if they experience discomfort or distress. This study involved no interventions or experimental procedures. In accordance with national and institutional data management regulations, all original research data and related documentation will be retained for at least five years following the conclusion of the primary study phase. Upon expiration of the retention period, all electronic data will be permanently and irreversibly deleted, whereas paper documents will undergo physical destruction by shredding.

### Data analysis

Data organization and analysis were performed via SPSS 27.0 and AMOS 26.0 statistical software. Count data were expressed as counts and percentages via SPSS 27.0, whereas continuous data were described as the mean ± standard deviations after normality testing. Pearson correlation analysis was employed to examine the relationships among nurses’ work stress, positive self-belief, and humanistic nursing competence. Multicollinearity diagnostics were performed on the predictor variables by calculating variance inflation factors (VIFs). All predictor variables exhibited VIF values of 1.269, which is significantly below the critical threshold of 10. This indicates that no severe multicollinearity issues exist. The Harman single-factor test was applied to all variables to detect common method bias. All the quantitative data in this study conformed to a normal distribution (with all the variables’ skewness and kurtosis coefficients within acceptable ranges: skewness < |2| and kurtosis < |7|), supporting the use of maximum likelihood estimation for model construction. AMOS 26.0 was used to construct a structural equation model to explore the path effects of work stress and humanistic nursing competence on positive self-belief among clinical nurses, with the significance level for path coefficient tests set at *P* < 0.05. Model fit was assessed via indicators such as χ²/df, CFI, TLI, and RMSEA. The specific criteria were as follows: χ²/df < 5 indicated acceptable fit, and < 3 indicated good fit; CFI and TLI > 0.90 indicated acceptable fit, and > 0.95 indicated good fit; RMSEA < 0.08 indicated reasonable fit, and < 0.05 indicated good fit. Mediation effects were further examined via the nonparametric bootstrap method, with 5000 repeated samples drawn from the original data to generate bias-corrected 95% confidence intervals. The mediation effect is considered established if this confidence interval does not include zero. Throughout the process, standardized estimates and their significance levels for direct, indirect, and total effects were reported concurrently.

## Results

### Characteristics of participants

Among the 542 nurses surveyed, the ages ranged from 22 to 54 years, with years of service spanning from 1 to 29 years. The cohort comprised 408 female nurses and 134 male nurses. Their places of work were distributed as follows: 198 in northern Anhui, 166 in the central region, and 178 in the southern region. With respect to educational qualifications, 100 held vocational school diplomas, 132 held college diplomas, 225 held bachelor’s degrees, and 85 held master’s degrees or higher. Within China’s professional title system, 134 were nurses, 240 were nurse practitioners, 101 were nurse supervisors, 35 were co-chief superintendent nurses, and 32 were chief superintendent nurses. With respect to marital status, 215 individuals were unmarried, and 327 were married (including those who had remarried or were widowed). Concerning current departmental placement, 222 nurses worked in internal medicine, 172 in surgery, 51 in gynecology, 36 in pediatrics, and 61 in critical care departments. Table 1 contains their detailed data.

**Table 1:** Characteristics of the participants (*n* = 542)

Variable	Category	Frequency (*n*)	Percentage (%)
Gender	Male	134	24.7
	Female	408	75.3
Age	< 30	157	29.0
	30 ~ 39	243	44.8
	40 ~ 49	125	23.1
	> 50	17	3.1
Working age	0 ~ 10	331	61.1
	11 ~ 20	184	33.9
	21 ~ 30	27	5.0
Professional title	Nurse	134	24.7
	Nurse Practitioner	240	44.3
	Nurse Supervisor	101	18.6
	Co-chief superintendent nurse	35	6.5
	Chief superintendent nurse	32	5.9
Marital status	Unmarried	215	39.7
	Married (including remarried and widowed)	327	60.3
Educational qualifications	Secondary technical school	100	18.5
	Junior college	132	24.4
	Undergraduate	225	41.5
	Master’s degree or above	85	15.7
Grade of hospital	Grade III Level A hospitals	263	48.5
	Grade III Level B hospitals	98	18.1
	Grade III Level C hospitals	68	12.5
	Grade II hospitals	72	13.3
	Grade I hospitals	41	7.6
Location	Northern Anhui	198	36.5
	Central Anhui	166	30.6
	Southern Anhui	178	32.9
Current Department	Internal Medicine	222	41.0
	Surgery	172	31.7
	Obstetrics & Gynecology	51	9.4
	Pediatrics	36	6.6
	Emergency Department and Intensive Care Unit	61	11.3

### Clinical nurses’ humanistic nursing competence, work stress, positive self-belief total score and dimension scores

Table 2 shows the total scores and subscale average scores for humanistic nursing competence, work stress, and positive self-belief among the 542 clinical nurses who participated in the study. The nurses’ work stress score was 78.54 ± 24.10, the humanistic nursing competence total score was 69.83 ± 11.47, and the positive self-belief total score was 72.94 ± 18.24.

**Table 2:** Clinical nurses’ humanistic nursing competence, work stress, and positive self-belief scores (*n* = 542)

Dimension	Number of items	Min	Max	Score (Mean ± SD)	Subscale averages (Mean ± SD)
**Nurses’ Work Stress**	35	35	140	78.54 ± 24.10	2.24 ± 0.69
Workload and time allocation	5	5	20	12.16 ± 4.41	2.43 ± 0.88
Working environment and resources	3	3	12	6.73 ± 2.64	2.24 ± 0.88
Patient care	11	11	44	23.34 ± 8.85	2.12 ± 0.80
Nursing specialization and employment	7	7	28	15.77 ± 5.68	2.63 ± 0.95
Management and interpersonal relations	9	9	36	20.73 ± 8.83	2.30 ± 0.98
**Humanistic Nursing Competence**	19	24	95	69.83 ± 11.47	3.68 ± 0.60
Disposition to Optimism	3	4	15	12.90 ± 2.46	4.30 ± 0.82
Sociability	3	3	15	12.82 ± 2.46	4.27 ± 0.82
Emotional Understanding	3	3	15	12.60 ± 2.73	4.20 ± 0.91
Self-efficacy	5	6	25	20.67 ± 4.73	4.13 ± 0.95
Affection	5	5	25	10.85 ± 4.49	2.17 ± 0.90
**Positive Self-Belief**	24	17	96	72.94 ± 18.24	3.04 ± 0.76
Character	4	1	16	12.38 ± 3.29	3.09 ± 0.82
Mastery	7	2	28	21.07 ± 5.66	3.01 ± 0.81
Strength	8	2	32	24.41 ± 6.36	3.05 ± 0.80
Enjoyment	5	1	20	15.15 ± 3.94	3.03 ± 0.79

### Comparative analysis of clinical nurses’ work stress, positive self-beliefs, and humanistic nursing competence scores across different characteristics

This study conducted a univariate analysis of work stress, positive self-belief, and humanistic nursing competence among 542 clinical nurses who participated in the survey. Research findings indicate that nurses with different genders, professional titles, and educational qualifications exhibit significant differences in scores for positive self-belief and humanistic nursing competence (*P* < 0.05). Nurses at varying levels of working age show significant differences in scores for work stress, positive self-belief, and humanistic nursing competence (*P* < 0.05). However, no significant differences were observed in scores for work stress, positive self-belief, or humanistic nursing competence among nurses with different marital statuses (*P* > 0.05). Specific data are presented in Table 3.

**Table 3:** Comparison of Clinical Nurses’ Work Stress, Positive Self-Belief, and Humanistic Nursing Competence Scores Across Different Characteristics (*n* = 542)

Variable	Nurses’ Work Stress (Mean ± SD)	Humanistic Nursing Competence (Mean ± SD)	Positive Self-Belief (Mean ± SD)
**Gender**			
Male	78.71 ± 23.60	76.93 ± 15.79	71.48 ± 9.18
Female	78.48 ± 24.29	71.63 ± 18.81	69.29 ± 12.09
t/F	0.096^a^	**3.210** ^**a**^	**2.206** ^**a**^
* P*	0.923	**0.001**	**0.028**
**Working age**			
0 ~ 10	80.87 ± 24.45	72.36 ± 18.63	68.82 ± 11.58
11 ~ 20	74.30 ± 22.34	72.98 ± 18.44	71.04 ± 11.19
21 ~ 30	78.78 ± 28.06	79.78 ± 8.37	73.85 ± 10.65
t/F	**4.726** ^**b**^	**7.935** ^**b**^	**4.006** ^**b**^
* P*	**0.012**	**< 0.001**	**0.019**
**Professional title**			
Nurse	78.86 ± 25.35	73.09 ± 17.01	69.22 ± 10.25
Nurse Practitioner	79.91 ± 23.01	70.74 ± 20.41	68.62 ± 11.94
Nurse Supervisor	75.68 ± 23.63	72.45 ± 17.43	71.27 ± 12.88
Co-chief superintendent nurse	74.71 ± 22.31	81.00 ± 10.08	72.66 ± 8.81
Chief superintendent nurse	80.06 ± 29.78	81.63 ± 8.54	73.81 ± 9.02
t/F	0.850^**b**^	**11.270** ^**b**^	**2.692** ^**b**^
* P*	0.496	**0.001**	**0.030**
**Marital status**			
Unmarried	79.30 ± 24.51	71.53 ± 18.92	69.08 ± 10.42
Married (including remarried and widowed)	78.03 ± 23.85	73.87 ± 17.75	70.32 ± 12.10
t/F	0.597^a^	-1.468^a^	-1.234^a^
* P*	0.551	0.143	0.218
**Educational qualifications**			
Secondary technical school	75.33 ± 22.16	66.82 ± 23.10	67.47 ± 12.72
Junior college	76.77 ± 24.24	74.08 ± 18.65	69.38 ± 10.75
Undergraduate	81.74 ± 24.73	72.86 ± 16.04	69.44 ± 12.30
Master’s degree or above	76.56 ± 23.78	78.59 ± 14.36	74.34 ± 6.68
t/F	2.365^**b**^	**6.422** ^**b**^	**11.799** ^**b**^
* P*	0.072	**0.001**	**0.001**

Note: a: t-tests, b: ANOVA

### Correlation analysis of nurses’ work stress, humanistic nursing competence, and positive self-belief

Pearson correlation analysis and correlation effect size interpretation guidelines [30] revealed that in this group of clinical nurses, work stress was moderately negatively correlated with humanistic nursing competence (*r* = -0.426, *P* < 0.001), while positive self-belief was strongly positively correlated with humanistic nursing competence (*r* = 0.629, *P* < 0.001). Additionally, work stress was moderately negatively correlated with positive self-belief (*r* = -0.460, *P* < 0.001). Additional details are provided in Table 4.

**Table 4:** Correlation analysis between humanistic nursing competence for nurses’ work stress, and positive self-belief (*n*=542, r-value)

Variable	1	2	3	4	5	6	7	8	9	10	11	12	13	14	15	16	17
**1**	1																
2	0.859**	1															
3	0.761**	0.609**	1														
4	0.728**	0.586**	0.570**	1													
5	0.758**	0.540**	0.453**	0.546**	1												
6	0.823**	0.692**	0.586**	0.510**	0.363**	1											
**7**	0.460**	-0.426**	0.381**	0.319**	0.350**	0.361**	1										
8	0.420**	-0.400**	0.343**	0.273**	0.308**	0.337**	0.952**	1									
9	0.462**	-0.429**	0.380**	0.334**	0.344**	0.365**	0.968**	0.886**	1								
10	0.438**	-0.398**	0.370**	0.310**	0.339**	0.338**	0.926**	0.832**	0.867**	1							
11	0.409**	-0.368**	0.339**	0.281**	0.337**	0.309**	0.925**	0.850**	0.868**	0.826**	1						
**12**	0.426**	-0.374**	0.324**	0.307**	0.305**	0.387**	0.629**	0.578**	0.632**	0.575**	0.582**	1					
13	0.401**	-0.381**	0.317**	0.303**	0.263**	0.343**	0.656**	0.611**	0.644**	0.610**	0.609**	0.868**	1				
14	0.362**	-0.325**	0.301**	0.281**	0.232**	0.332**	0.626**	0.593**	0.617**	0.570**	0.578**	0.860**	0.850**	1			
15	0.378**	-0.361**	0.332**	0.262**	0.199**	0.374**	0.596**	0.569**	0.598**	0.540**	0.526**	0.857**	0.819**	0.849**	1		
16	0.381**	0-0.338**	0.344**	0.262**	0.237**	0.360**	0.655**	0.611**	0.653**	0.599**	0.599**	0.898**	0.866**	0.867**	0.861**	1	
17	-0.041	0.007	0.075	0.029**	0.137**	-0.014	0.147**	0.172**	0.127**	0.136**	0.113**	0.143**	0.246**	0.246**	0.240**	0.231**	1

Note：**P<0.01 1: Nurses' Work Stress 2: Nursing specialisation and employment 3: Workload and time allocation 4: Working environment and resources 5: Patient care 6: Management and interpersonal relations 7: Positive Self-Belief 8: Mastery 9: Strength 10: Enjoyment 11: Character 12: Humanistic Nursing Competence 13: Disposition to Optimism 14: Sociability 15: Emotional Understanding 16: Self-efficacy 17: Affection

### Analysis of the mediating effect of positive self-belief on the relationship between nursing work stress and humanistic nursing competence

#### Common method bias test

The common method bias test was conducted via Harman’s single-factor test. The results revealed eight factors with eigenvalues exceeding 1. The first principal component obtained before rotation accounted for 31.01% of the total factor loadings, falling below the 40% critical threshold. This finding indicates that no significant common method bias exists in this study [31].

### Analysis of the mediating effect of positive self-belief on the relationship between clinical nurses’ work stress and humanistic nursing competence

**Fig. 1:**
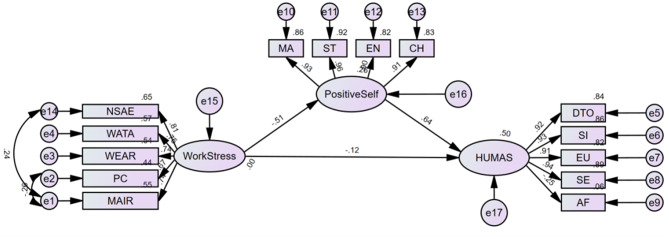
Mediating effect model of positive self-belief. Note: *NSAE* Nursing specialization and employment, *WATA* Workload and time allocation, *WEAR* Working environment and resources, *PC* Patient care, *MAIR* Management and interpersonal relations, *MA* Mastery, *ST* Strength, *EN* Enjoyment, *CH* Character, *DTO* Disposition to optimism, *SI* Social Interaction, *EU* Emotional Understanding, *SE* Self-efficacy, *AF* Affection

To control for the influence of potential confounding factors, this study incorporated demographic variables such as gender, years of service, educational attainment, professional title, and marital status as covariates within the structural equation model. In the model constructed using AMOS 26.0 software, these variables were all set as exogenous observed variables, with their paths to the three core latent variables (work pressure, positive self-belief, and humanistic care ability) freely estimated. Consequently, the final model fit indices, standardized path coefficients, and mediation effect results were statistically controlled for these covariates. This study employed clinical nurses’ work stress as the independent variable, positive self-belief as the mediating variable, and humanistic nursing competence as the dependent variable. Structural equation modeling was conducted via AMOS 26.0 software, with model modifications implemented by adding covariance paths based on model fit indices. The final χ²/df value was 2.275, with a comparative fit index (CFI) of 0.986, a threshold-limit index (TLI) of 0.985, and a root mean square error of approximation (RMSEA) of 0.051. The model demonstrated an ideal fit to the data. When bootstrap sampling with 5,000 iterations was used to test the mediating effect, the calculated 95% confidence interval for the mediating effect was − 0.145 to -0.083 (excluding zero), indicating a statistically significant difference (*P* < 0.001). This confirms that positive self-belief mediates the relationship between clinical nurses’ work stress and humanistic nursing competence, accounting for 73.33% of the total effect (Fig. 1; Table 5).

**Table 5:** Bootstrap analysis of path effect significance testing (*n* = 542)

Effect relationship	Path situation	Boot SE	95% CI	*P*	Proportion of effect size (%)
Total effect	-0.155	0.021	-0.195ཞ-0.114	< 0.001	100.00
Direct effect	-0.041	0.015	-0.074ཞ-0.014	0.006	26.67
Indirect effect	-0.113	0.016	-0.145ཞ-0.083	< 0.001	73.33

## Discussion

The cultivation of positive self-beliefs among clinical nurses is closely linked to their stress coping abilities and enhanced humanistic nursing competence. Based on self-determination theory, this study examined the interrelationships among work stress, positive self-beliefs, and humanistic nursing competence, while testing the mediating role of positive self-beliefs. Findings revealed a psychological pathway through which positive self-beliefs mediate the relationship between work stress and humanistic nursing competence, providing theoretical foundations for developing targeted intervention strategies.

### Current status of humanistic nursing competence among Chinese nurses

This study found that Chinese nurses’ humanistic nursing competence scores (69.83 ± 11.47) were at a moderately low level, similar to findings from Chinese scholar Liu Shenmei’s research [32] (surveying 582 participants with an overall humanistic nursing competence score of 69.05 ± 10.04). This phenomenon may be related to the emphasis on nursing procedural skills in Chinese nursing education, which has relatively neglected the cultivation of humanistic care [33]. Simultaneously, Chinese nurses commonly face dual pressures from both clinical duties and managing tasks. This dual burden can heighten professional frustration and diminish work enthusiasm among nurses, potentially limiting their opportunities to develop humanistic care qualities, awareness, and competencies in clinical practice. Ultimately, this undermines their capacity for humanistic nursing. This high-stress work environment constitutes the type of situation highlighted by self-determination theory, one that may undermine the fulfillment of nurses’ fundamental psychological needs. Research indicates [34] that among Chinese nurses’ sources of work stress, “management-level task pressure” accounts for 38.6%, significantly higher than the 29.3% reported by Western nursing populations. This disparity reflects differences in nursing management styles and organizational cultures between Eastern and Western contexts. Therefore, enhancing Chinese nurses’ humanistic care capabilities requires systematic interventions focused on optimizing supportive professional environments and deepening humanistic care education.

### The mediating role of positive self-belief between work stress and humanistic nursing competence

This study found that work stress was not only moderately negatively correlated with positive self-belief but also moderately negatively correlated with humanistic nursing competence (a finding similar to that reported among Korean nurses [35] (*r* = -0.305, *P* < 0.001)), thereby validating Hypothesis 1 and Hypothesis 2. According to self-determination theory, high work stress constitutes an impeding environment that systematically undermines nurses’ three fundamental psychological needs—autonomy, competence, and relatedness. This occurs by restricting their professional autonomy, continually challenging their professional competence [36], and encroaching upon the time and energy required to establish deep relationships with patients. This prolonged frustration of needs may impact nurses’ psychological and behavioral functioning in two ways: On one hand, it may undermine nurses’ positive self-beliefs regarding their professional role efficacy and developmental potential. As a deep, integrated form of self-awareness, the formation and maintenance of positive self-beliefs depend precisely on the sustained fulfillment of basic psychological needs. This aligns with previous research [37]; on the other hand, persistent frustration in meeting demands may erode the intrinsic motivation required for nurses to engage in high-emotional, high-empathy care practices, thereby diminishing their capacity for humanistic nursing.

The study also found a significant positive correlation between positive self-belief and humanistic nursing competence, validating Hypothesis 3. Humanized care is a practice driven by intrinsic motivation and characterized by high emotional investment. When nurses possess stable positive self-beliefs, they are more likely to stimulate and sustain their intrinsic motivation. This enables them to effectively mobilize psychological resources even under stress, thereby exhibiting stronger empathy and proactive care behaviors [38]. Within China’s healthcare culture that emphasizes collective dedication, positive self-belief manifests as a deep conviction that one can make a substantive contribution to patient well-being through professional nursing care. This belief serves as a vital internal driving force for sustaining the capacity for humanistic care.

The core finding of this study is that positive self-belief partially mediates the relationship between nurses’ work stress and humanistic nursing competence, explaining 73.33% of the total effect, thereby supporting Hypothesis 4. This provides empirical support for the application of self-determination theory in nursing and reveals specific psychological pathways. When nurses operate in an obstructive environment characterized by high work stress, their fundamental psychological needs become compromised: Nurses experience dual pressures from both clinical duties and managing tasks, preventing them from providing patient-centered care according to their own will. They feel unable to meet additional work demands, thereby undermining their sense of autonomy and competence. Simultaneously, heavy workloads leave insufficient time for relationship-building and communication with patients, damaging their sense of belonging. Positive self-belief is the concentrated manifestation of psychological fulfillment after basic psychological needs are met. When persistent pressure undermines nurses’ fundamental psychological needs, their positive self-belief in their professional value becomes shaken. This erodes their intrinsic motivation to proactively provide emotional care and deliver humanistic nursing, ultimately leading to a decline in their capacity for humanistic care. A cross-sectional study suggests that positive psychological resources, such as self-efficacy, can serve as a buffer between stress and job performance [39]. However, it is worth noting that, unlike self-efficacy, which is focused on confidence in accomplishing particular tasks [40], positive self-belief constitutes a fundamental affirmation of one’s self-worth, capabilities, and developmental potential, possessing greater foundational stability. It is embedded within the care-oriented values unique to the nursing profession, thereby providing a deeper psychological impetus for humanistic nursing. These findings suggest that while administrators strive to improve nurses’ working environments and effectively alleviate stress, they should place particular emphasis on cultivating and strengthening nurses’ positive self-beliefs. By systematically enhancing this critical psychological resource, the impact of work-related stress can be more effectively buffered, thereby fundamentally preserving and enhancing their capacity for humanistic care.

### Theoretical implications: validation and extension of self-determination theory

The findings of this study not only validate the applicability of self-determination theory in the nursing field but also significantly deepen and expand upon it through contextualized research.

First, the findings of this study empirically validate the core mechanisms of self-determination theory within nursing practice. The research reveals that the pathway of work stress (impeding environment)→depletion of positive self-beliefs (motivational resources)→diminished person-centered nursing competence (behavioral function) substantiates the theory’s central mechanism: the social environment regulates intrinsic motivation and behavior by influencing the fulfillment of individuals’ fundamental psychological needs. This provides strong empirical support for the applicability of this theory in nursing, a profession with high emotional demands. More importantly, by introducing the mediating variable of positive self-belief, this study provides a significant refinement to the motivational resources dimension of self-determination theory. Within the framework of self-determination theory, intrinsic motivation is a relatively broad concept. This study operationalizes it in the nursing field as a positive self-belief deeply integrated with professional roles and oriented toward humanistic care. This construct emphasizes a fundamental affirmation of one’s professional value and competence, serving not only as a manifestation of motivation but also as a stable resource for professional identity. This refines our understanding of what specific internal psychological resources drive professional behavior within specialized fields. Furthermore, this study expands the application of self-determination theory within the field of emotional labor [41]. The findings indicate that in emotionally demanding professions such as nursing, the key to sustaining high-quality humanistic care behaviors may lie in cultivating a positive professional self-concept that effectively mitigates environmental stressors and protects intrinsic motivation. This offers a new perspective for developing interventions based on self-determination theory: shifting from merely improving external environments to simultaneously focusing on preserving and enhancing individuals’ internal psychological resources that integrate with their occupational roles.

Although this study proposes the pathway “work stress → positive self-belief → humanistic nursing competence” based on theory, the cross-sectional design limits the determination of causal direction. We carefully considered other possible theoretical explanations. First, reverse causality may exist, where greater humanistic nursing competence enhances nurses’ positive self-belief by improving their professional accomplishment and job satisfaction, thereby buffering their perceived work stress. Second, positive self-belief may function as a relatively stable personal trait, simultaneously influencing nurses’ assessment of work stress and their exercise of humanistic nursing competence. Finally, unmeasured confounding variables (such as the organizational support climate or personal resilience) may concurrently affect all three variables. These alternative explanations require further validation through longitudinal research designs.

### Strengths

This study reveals two potential implications for experimentation. First, for the nursing population, the research identifies a significant association between work stress levels, positive self-belief, and humanistic nursing competence. This provides a preliminary theoretical foundation for enhancing humanistic nursing competence through stress regulation and cultivating positive self-belief. Second, in terms of management practices, the results demonstrate a positive correlation between nurses’ positive self-belief and their humanistic nursing competence, offering new perspectives for managers in human resource development. For example, regular stress assessments and targeted interventions to improve work experiences, coupled with deliberate cultivation of positive self-beliefs, may positively impact overall nursing service quality. Notably, owing to the limitations of the cross-sectional study design, the directional relationships among variables require further validation through longitudinal research.

### Limitations

First, the cross-sectional study design employed here precludes determining the direction of causality among variables. For example, reverse or bidirectional effects may exist between work stress, positive self-beliefs, and humanistic nursing competencies. Second, data derived from self-report questionnaires may be subject to common method bias and social desirability effects. Furthermore, despite including basic demographic variables, important covariates (e.g., departmental atmosphere, leadership management style, individual psychological resilience) may have been omitted, potentially influencing core variable relationships. Regarding sampling methods, convenience sampling and geographical limitations (restricted to Anhui Province) also constrained sample representativeness and the generalizability of the findings. Future research may focus on comparing nurse cohorts with distinct demographic characteristics—such as varying educational backgrounds, years of experience, or clinical departments—to test the generalizability of this study’s mediation model or identify potential moderating effects. This would aid in developing more targeted intervention strategies. Future research could adopt longitudinal or experimental designs, collect data through multicenter random sampling, and combine objective measures with self-reported data to more comprehensively examine causal pathways and underlying mechanisms among variables.

## Conclusion

This study examined the relationships among positive self-belief, work stress, and humanistic nursing competence among clinical nurses in China, providing a theoretical basis for the interactions among these three constructs. These findings confirmed that positive self-belief partially mediates the relationship between clinical nurses’ work stress and their humanistic nursing competence. These findings offer reference points for subsequent exploratory and intervention studies. Research should subsequently examine the external factors influencing nurses’ positive self-belief and work stress in multiple dimensions, including occupational burnout, organizational culture, and leadership styles. Consideration should also be given to implementing targeted interventions to enhance nurses’ humanistic nursing competence. Furthermore, adopting a mixed-methods approach combining quantitative and qualitative research could yield more comprehensive insights. Exploring variations among nurses across different regions would increase the specificity of research conclusions.

### Implications and recommendations

This study preliminarily reveals the intrinsic relationships among work stress, positive self-beliefs, and humanistic nursing competence among Chinese nursing staff, offering new insights into the formation mechanisms of humanistic nursing competence within the Chinese nursing context. Based on the findings, a systematic intervention framework is proposed across three levels. At the nursing management practice level, implementing evidence-based multidimensional intervention programs is recommended. Specifically, group counseling centered on cognitive behavioral therapy (CBT) for positive self-beliefs (once weekly, 90 min per session, for 8 weeks) can be conducted to help nurses establish positive professional cognitive schemas. Concurrently, strength-based mindfulness-based stress reduction training (twice weekly, 60 min per session, for 6 weeks) should be implemented to enhance nurses’ emotional regulation capabilities. With respect to organizational support, scientific nurse-to-patient ratio standards should be established to ensure that nursing workloads remain within reasonable limits. Flexible scheduling systems should be implemented to guarantee adequate rest and recovery time for nurses. Create “psychological mutual aid circles” at the department level, conducting regular team-building activities to foster a supportive work environment. These measures aim to reduce nurse turnover and mitigate occupational burnout. At the policy formulation level, relevant departments should incorporate humanistic nursing competencies into core nursing quality evaluation metrics and make nurses’ mental health status a key indicator in healthcare institution assessments. Concurrently, the development of the Nursing Human Resource Allocation Standards should be accelerated to ensure scientifically sound nurse-to-patient ratios. Nursing education accreditation standards should explicitly mandate positive psychology cultivation and humanistic nursing competence training as required coursework, ensuring systematic skill development during students’ academic training. With respect to nursing education reform, nursing institutions should introduce a “Positive Psychology and Humanistic Care” curriculum series, integrating cognitive behavioral training, mindfulness-based stress reduction, and communication skills into the teaching framework. Teaching methods such as clinical scenario simulations, reflective writing, and compassionate practice journals should help students develop positive professional identities and enhance their psychological resilience in facing clinical challenges. This curriculum should comprise no fewer than 36 credit hours and be incorporated into graduation requirements. Additionally, healthcare institutions should establish electronic document template libraries. Standardized assessment forms and automated documentation systems can reduce the proportion of nonclinical tasks, enabling nurses to dedicate more time to direct patient care. These systematic, multilevel interventions not only effectively alleviate nursing work stress and strengthen positive self-beliefs but also fundamentally enhance the quality of humanized care. This lays a solid foundation for building a people-centered, high-quality nursing service system.

## Data Availability

All data involved in this study can be obtained from the corresponding author upon reasonable request.
